# The Osseous Pathology of Purpura Fulminans in a Two-Year-Old Child: A Case Report

**DOI:** 10.5704/MOJ.2303.021

**Published:** 2023-03

**Authors:** S Mohd-Razali, K Ahmad-Affandi, S Ibrahim, AH Abdul-Rashid, N Abdul-Shukor

**Affiliations:** 1 Department of Orthopaedics and Traumatology, Universiti Kebangsaan Malaysia, Kuala Lumpur, Malaysia; 2 Department of Pathology, Universiti Kebangsaan Malaysia, Kuala Lumpur, Malaysia

**Keywords:** purpura fulminans, disseminated intravascular coagulation, gangrene, amputation

## Abstract

Purpura fulminans (PF) is a severe clinical manifestation of *Neisseria meningitides* infection that is associated with high mortality rates in children. Survivors are frequently left with debilitating musculoskeletal sequelae. There is a paucity of reports on the musculoskeletal pathology of purpura fulminans. We report on a 2-year-old boy with purpura fulminans due to meningococcemia. The child developed distal gangrene in both the upper and lower limbs. Amputations were done for both lower limbs. Histological examination of the amputated specimens showed an inflammatory process and features of osteonecrosis. The latest follow-up at the age of 6 years showed a right knee valgus due to asymmetrical growth arrest of the proximal tibia. PF and its complications are challenging to treat and may require a multidisciplinary approach to improve patient’s functional ability.

## Introduction

Purpura fulminans (PF) is a severe clinical manifestation of fulminant meningococcemia caused by *Neisseria meningitidis*. The causative gram-negative diplococcus is transmitted via respiratory secretions and is associated with mortality rates as high as 15-30% in children^1,2^.

The thrombotic syndrome of PF is manifested as disseminated intravascular coagulopathy (DIC) and vascular thrombosis leading to cutaneous and musculoskeletal complications^1-3^. The incidence and management of the orthopaedic sequelae namely amputations, growth plate arrest, osteonecrosis, and limb length discrepancy (LLD) are variable and have been well described in the literature^1-3^. However, there is a paucity of reports in the literature on the gross and histopathology of the orthopaedic complications of purpura fulminans. To the best of our knowledge, there have been only two previous studies describing the osseous histopathology of purpura fulminans^4,5^.

The aim of our case report is to highlight the gross pathology and histopathology of purpura fulminans affecting the musculoskeletal system in a two-year-old boy as well as the challenges in managing its complication.

## Case Report

A two-year-old boy presented with a short history of fever and erythematous patches over the trunk and both upper and lower limbs. The infection quickly escalated to septic shock with multi-organ failure. Blood cultures grew *Neisseria meningitides* and the patient was treated with antibiotics and supportive care in another hospital. Gangrene occurred in both the upper and lower limbs within a few days.

**Fig. 1:**
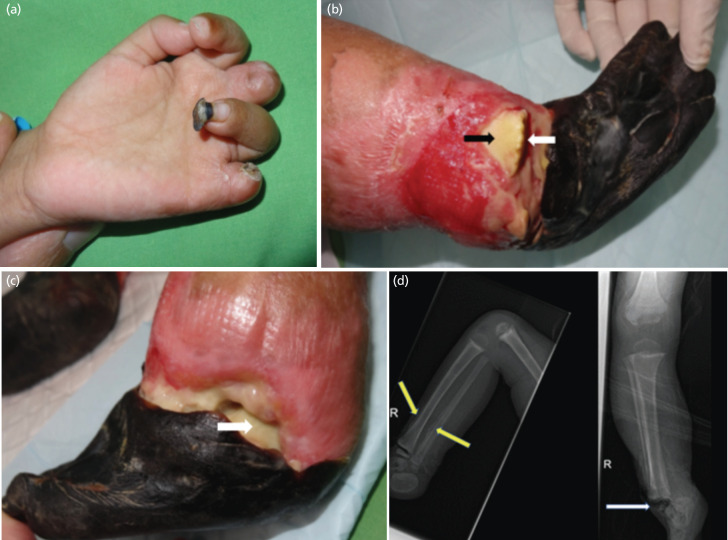
(a) Auto-amputation of the middle and little fingers. Dry gangrene at the tip of the ring finger. (b) Gangrene of the right foot. Necrosis of the physis resulted in the gap (white arrow) distal to the metaphysis (black arrow) of the right tibia. (c) Gangrene of the left foot. Soft tissue necrosis has exposed the ankle joint. The talus is visible (arrow). (d) Plain radiographs showing periosteal elevation of both tibia and fibula (yellow arrow). There is separation of the epiphysis from the metaphysis of the right tibia (white arrow).

The child was transferred to our hospital after three weeks of being treated at the primary hospital to stabilise his general condition. Auto-amputation of the left index and ring fingers had occurred (Fig. 1a). Both feet were gangrenous. The distal metaphysis of the right tibia was exposed due to necrosis of the overlying skin. Necrosis of the distal tibial physis resulted in separation of the epiphysis from the metaphysis (Fig. 1b). The left ankle joint and talus was visible due to necrosis of the overlying soft tissue (Fig. 1c). Plain radiographs showed periosteal elevation of both tibia and fibula with separation of the epiphysis from the metaphysis of the right tibia (Fig. 1d). Radiographs of the left tibia showed similar bony changes but without physeal changes.

**Fig. 2:**
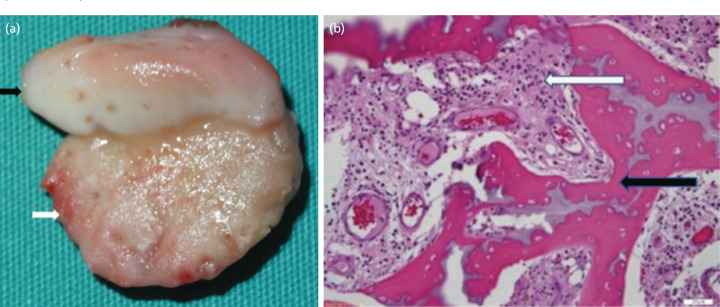
(a) Remnant of the physis (black arrow) attached to the epiphysis (white arrow) of the right distal tibia. (b) The metaphysis of the right tibia shows osteonecrosis of the bony trabeculae (black arrow) and granulation tissue formation with marked acute inflammatory infiltrates (white arrow) within the marrow spaces (H&E 200X).

The child was treated with a transtibial amputation just proximal to the right ankle and a left ankle disarticulation. The gross pathology of the amputated right lower limb showed a remnant of the physis still attached to the epiphysis (Fig. 2a).

Biopsies were obtained from the distal epiphysis of both right tibia and fibula, distal metaphysis of right tibia and the left talus. Histopathological examination of the right tibia metaphysis shows osteonecrosis of the bony trabeculae and granulation tissue formation with marked acute inflammatory infiltrates within the marrow spaces (Fig. 2b).

**Fig. 3:**
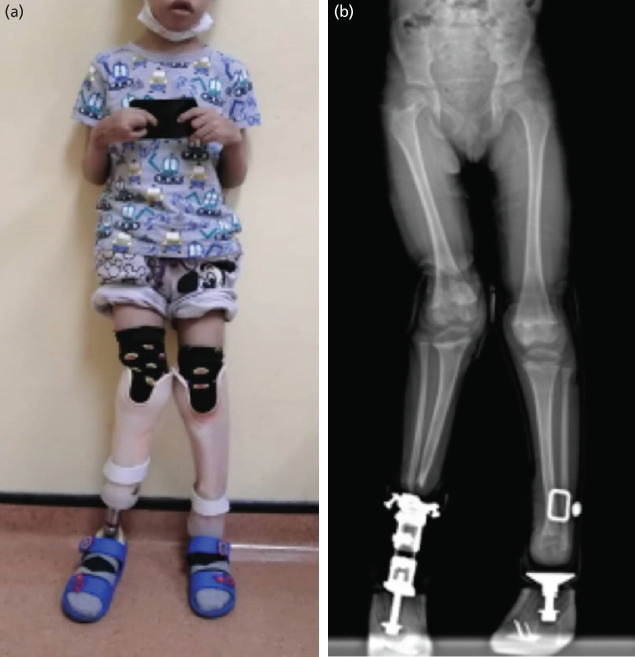
(a) The child at age six years with bilateral below knee prosthesis. (b) The radiograph shows a valgus deformity of the right knee due to asymmetrical growth arrest of the proximal tibia. Physeal irregularities are also seen proximally and distally in the right femur, left distal femur and left distal tibia.

His latest follow-up at the age of six-year-old showed the child walking with bilateral below-knee prosthesis (Fig. 3a). He developed right knee valgus due to growth arrest of the proximal tibia (Fig. 3b) and is being closely followed-up, which may need corrective osteotomy later to realign the deformity.

## Discussion

Based on our literature review, there have been only two previous reports on the osseous histopathology of purpura fulminans^4,5^. Grogan *et al*^4^ described the biopsy specimens from 9 children with meningococcemia while Campbell *et al*^5^ described the biopsy specimens of a 42-year-old woman with meningococcemia.

PF is primarily a thrombotic event. The histopathologic processes occurring in PF have been linked to local Shwartzman reactions, which are initiated by endotoxins released by gram negative organisms. Perivascular infiltrates of neutrophils and macrophages can be seen around vessels^1-5^. As seen in our patient, skin necrosis is much more common to occur at the periphery i.e. fingers and feet. There is a predilection for the lower limb as these are areas of the integument from which blood is shunted during the state of shock.

The basis of osteochondral injury is a widespread intraosseous coagulopathy affecting all layers of the bone including the growth plates, leading to osteonecrosis. Osteonecrosis can be seen in multiple histology sections taken from our patient. Irreversible osteocytic necrosis requires a minimum of two hours of complete ischemia with total anoxia. The acute ischaemia affects the cartilage-canal system between the growth plate and epiphyseal metaphyseal layer. Vessel occlusion and vascular thrombosis involved all layers of the growing bone in variable multi-foci patterns. An inflammatory response can also be observed in the marrow and sub-periosteal spaces^1-5^.

Grogan *et al*^4^ described the biopsies of bone and cartilage from nine children with meningococcemia resulting in gangrene of the distal parts of the limbs. The histological changes seen were consistent with acute osteomyelitis and ischaemia. Biopsies taken after revision of amputation several years later showed physeal bone bridges had developed as a result of ischaemia.

Campbell *et al*^5^ reported on a 42-year-old woman with meningococcemia who developed osteonecrosis – biopsies had been taken from the radius and tibia. Similarly, in our patient, osteonecrosis was seen in the epiphysis and metaphysis of the amputated distal tibia. A marked inflammatory response is evidenced by neutrophilic exudates and acute inflammatory infiltrates.

Late complications can present clinically in the form of limb length discrepancy and bowing of extremities. Radiological changes include beaking joints, lytic bone lesions and asymmetrical physeal destruction^1-5^. The physis may not appear to be involved early on, but with time the insult is quite evident. Growth arrests occur preferentially under areas of cutaneous scarring and is reported to be usually symmetrical^1-3^. However, our patient had developed a right knee valgus deformity due to asymmetrical growth arrest of the proximal tibia.

In conclusion, PF and its complications are challenging to treat. The pathological features of osteochondral injury provide the scientific basis for understanding musculoskeletal deformities in a surviving patient. Orthopaedic follow-up is mandatory to identify and manage the long-term sequelae of meningococcemia.
